# Engineering the hyperthermophilic archaeon *Pyrococcus furiosus* for 1-propanol production

**DOI:** 10.1128/aem.00471-25

**Published:** 2025-04-07

**Authors:** Hailey C. O'Quinn, Jason L. Vailionis, Tania N. N. Tanwee, Katherine S. Holandez-Lopez, Ryan G. Bing, Farris L. Poole, Ying Zhang, Robert M. Kelly, Michael W. W. Adams

**Affiliations:** 1 Department of Biochemistry and Molecular Biology, University of Georgia 174518, Athens, Georgia, USA; 2 Department of Cell and Molecular Biology, College of the Environment and Life Sciences, University of Rhode Island 118730, Kingston, Rhode Island, USA; 3 Department of Chemical and Biomolecular Engineering, North Carolina State University 242511 , Raleigh, North Carolina, USA; Kyoto University, Kyoto, Japan

**Keywords:** *Pyrococcus furiosus*, archaea, thermophile, metabolic engineering, 1-propanol, genome-scale modeling

## Abstract

Society relies heavily on chemicals traditionally produced through the refinement of fossil fuels. The conversion of renewable biomass to value-added chemicals by microbes, particularly hyperthermophiles (T_opt_ ≥80°C), offers a renewable alternative to this traditional approach. Herein, we describe the engineering of the hyperthermophilic archaeon *Pyrococcus furiosus*, which grows optimally (T_opt_) at 100°C, for the conversion of sugar to 1-propanol. This was accomplished by constructing a hybrid metabolic pathway consisting of two native and seven heterologously produced enzymes to convert acetyl-CoA from carbohydrate metabolism to 1-propanol. A total of eleven foreign genes from two other organisms were utilized, one from the thermophilic bacterium *Thermoanaerobacter* sp. strain X514 and 10 from the thermoacidophilic archaeon *Metallosphaera sedula,* both of which grow optimally near 70°C. The recombinant *P. furiosus* strain produced 1-propanol at similar concentrations (up to ~1 mM) when incubated at 75°C to activate the gene products of *Thermoanaerobacter* sp. strain X514 and *M. sedula* and by initially incubating at 95°C for *P. furiosus* growth and then subsequently returning to 75°C to promote 1-propanol formation. Note that 1-propanol was not produced if the culture was grown only at 95°C. This work has the potential for future optimization through harnessing the genome-scale metabolic model of *P. furiosus* that was used herein to identify engineering targets to increase 1-propanol titer.

**IMPORTANCE:**

As petroleum reserves become increasingly strained, the development of renewable alternatives to traditional chemical synthesis becomes more important. In this work, a high-temperature biological system for sugar to 1-propanol conversion was demonstrated by metabolic engineering of the hyperthermophilic archaeon *Pyrococcus furiosus* (T_opt_ 100°C). The engineered strain produced 1-propanol by temperature shifting from 75°C to 95°C and then back to 75°C to accommodate the temperature ranges for native and foreign proteins associated with 1-propanol biosynthesis. Genome-scale metabolic modeling informed the carbon and reductant flux in the system, identified potential factors limiting 1-propanol production, and revealed potential optimization targets.

## INTRODUCTION

The primary alcohol 1-propanol, also referred to as n-propanol, propyl alcohol, or simply propanol, is an important chemical in the modern industrial sector (1). This compound is widely used as a solvent or additive in a number of products, including resins, lacquers, polishing compounds, and adhesives and is also utilized for the production of ointments, lotions, and soaps in the pharmaceutical and cosmetics industries (2). 1-propanol also has potential as a high-energy biofuel, although this application is impacted by the high cost of synthesis. Current industrial production of 1-propanol is based on the refinement of petrochemicals, notably utilizing inorganic catalysts and often multiple levels of resource-intensive synthesis and distillation (1–4). This production method is ultimately reliant on the consumption of fossil fuels; hence, alternative and renewable methods to produce 1-propanol are desirable.

To date, biological production of 1-propanol has been limited; no organism natively produces 1-propanol in sufficient quantities to be considered industrially scalable. Genetic engineering approaches have been employed with *Propionibacterium freudenreichii* (5), *Escherichia coli* (6–8), *Corynebacterium glutamicum* (9), and *Saccharomyces cerevisiae* (10) for the production of 1-propanol. Of these, *E. coli* was reported to be the most productive, with batch cultures generating 33 mM 1-propanol (6), whereas a fed-batch reactor system yielded 178 mM (7). Much lower yields were reported with *C. glutamicum* (12 mM [9]), *P. freudenreichii* (8.3 mM [5]), and *S. cerevisiae* (0.45 mM [10]). Although these efforts have yielded the target product 1-propanol, they have not yet seen industrial application. Notably, these organisms are all mesophiles, growing at temperatures less than 40°C and thus face key scalability issues associated with mild temperature bioprocessing and the requirement for sterile conditions. To date, the highest growth temperature of an organism that has been engineered for 1-propanol production is 55°C by the moderately thermophilic aerobic bacterium *Thermobifida fusca,* which yielded 7.9 mM (11).

Although microbial bioprocessing efforts utilizing engineered organisms have been of great interest in recent decades, most industrial applications have remained focused on mesophilic organisms. However, the application of thermophiles (T_opt_ between 60°C-79°C) and hyperthermophiles (T_opt_ ≥80°C) for bioprocessing efforts has the potential to expand this field. High-temperature bioprocessing systems can offer unique benefits, including the reduction of bioreactor cooling costs, minimizing the risk of microbial and phage contamination, and increased solubility of key substrates (12–14). Additional hypothesized benefits include mid-growth purification of volatile chemicals and the ability to leverage unique native biochemistry and physiology of microbes that grow at higher temperatures (12).

*Pyrococcus furiosus* is a hyperthermophilic archaeon in the order Thermococcales originally isolated from a marine hydrothermal vent near Vulcano, Italy; it has an optimum growth temperature of 100°C with a growth range from 70°C–103°C (15, 16). *P. furiosus* is an obligate heterotroph, fermenting hexose disaccharides into carbon dioxide, hydrogen, and acetate as the only major products. A naturally competent *P. furiosus* strain, COM1, serves as the basis for the organism’s genetic system, making it the most thermophilic organism with such tools available (17). This allows homologous modifications to the native chromosome and stable integration of foreign constructs (17), leading to interest in *P. furiosus* as a platform for high-temperature production of bio-based chemicals. In order to make genomic insertions without disrupting the native chromosome, a DNA tiling array (18) identified regions of little to no transcriptional activity, or “genomic islands,” where insertions can be made. Using this system, *P. furiosus* has been engineered to produce non-native chemicals, such as lactate (19), 1-butanol (20, 21), and 3-hydroxypropionate (3-HP) (22).

**Fig 1:**
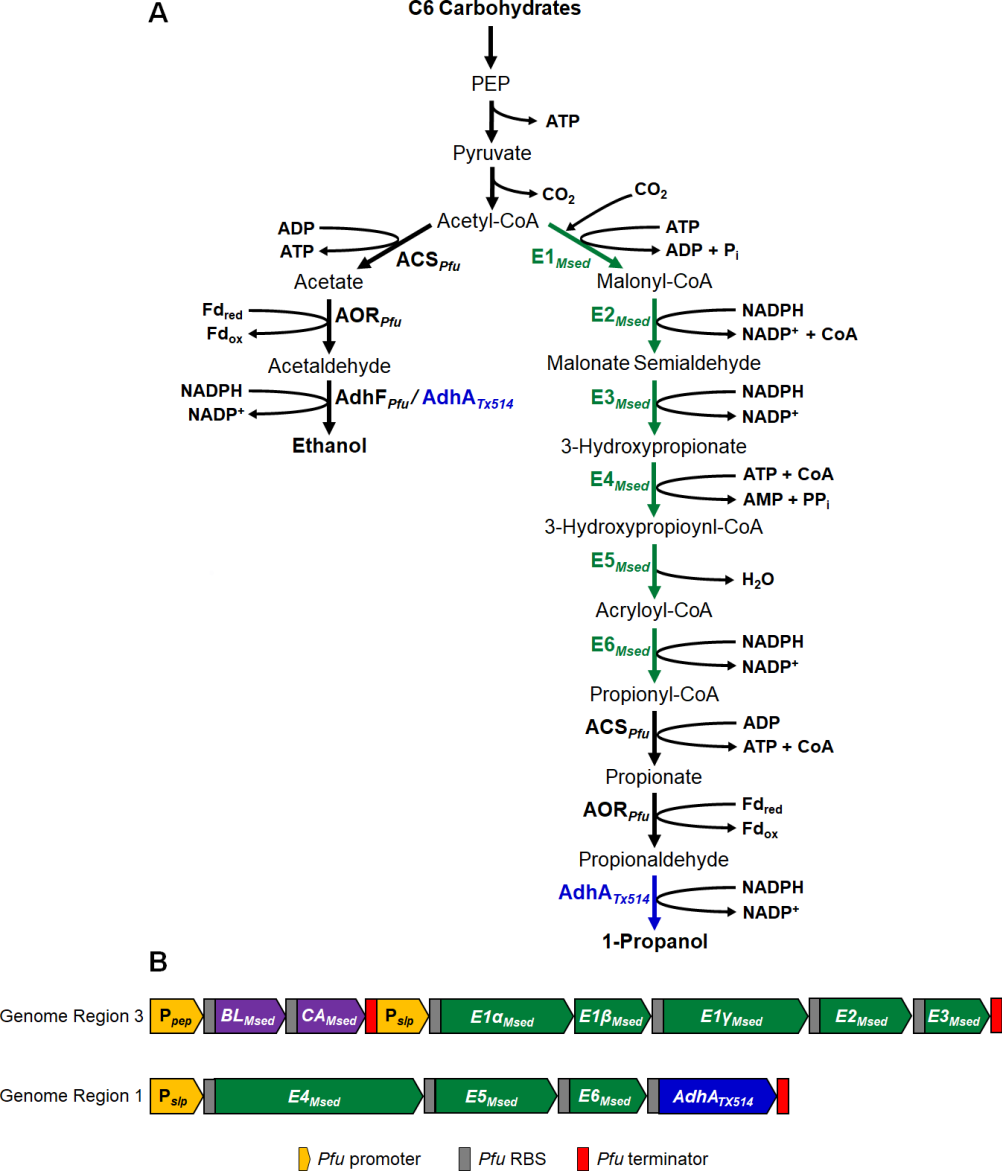
Metabolic pathways for target 1-propanol and off-target ethanol in *P. furiosus*. (A) 1-propanol pathway with redox chemistry (right) and off-target ethanol pathway (left). Reactions in green are sourced from *M. sedula* (*Msed*). Reactions in blue are sourced from *Thermanaerobacter* X514 (*TX514*). The metabolic pathway for 1-propanol production includes the following enzymes: E1 (acetyl-CoA/propionyl-CoA carboxylase), E2 (malonyl-CoA/succinyl-CoA reductase), E3 (malonate semialdehyde reductase), E4 (3-hydroxypropionate-CoA ligase), E5 (3-hydroxypropionyl-CoA dehydratase), E6 (acryloyl-CoA reductase), ACSII (acetyl-CoA synthase), AOR (aldehyde ferredoxin oxidoreductase), and AdhA (alcohol dehydrogenase). A branching pathway that leads to ethanol production is also shown, including ACS, AOR, and AdhA. (B) Constructs used in this study to express heterologous enzymes. Constructs are designed for chromosomal insertion into GR3 (top) and GR1 (bottom).

Production of 3-HP utilizes the 3-hydroxypropionate/4-hydroxybutyrate (3-HP/4-HB) carbon fixation cycle from the thermoacidophilic archaeon *Metallosphaera sedula*, incorporating three pathway enzymes to convert carbohydrate-derived acetyl-CoA to 3-HP (23–26). This pathway was optimized through the expression of two additional *M. sedula* enzymes, biotin protein ligase (BL) and carbonic anhydrase (CA) (25). Both BL and CA serve as accessory enzymes for the first enzyme in this pathway, acetyl-CoA carboxylase (E1, Fig. 1), serving to biotinylate E1 and hydrate CO_2_ to the bicarbonate for use in the reaction (25). Additionally, ethanol production has been significantly increased from low native concentrations through both the insertion of heterologous enzymes and the overexpression of the native ethanol-producing alcohol dehydrogenase (21, 27, 28). By growing engineered strains at sub-optimal temperatures, as low as 70°C, a wider range of enzymes becomes compatible with their functional expression in *P. furiosus*. In addition, a temperature shift strategy can be employed to balance growth and product formation through thermally decoupling these functions: cultures are grown near 100°C for the accumulation of biomass, then switched to a lower temperature to induce product formation. This approach has been used for temperature-dependent formation of lactate and 3-hydroxypropionate (19, 22).

The archaeon *Thermococcus onnurineus* (T_opt_ 80°C), another member of the Thermococcales order, was used for the first industrial application of a genetically engineered hyperthermophile in the conversion of waste CO from steel refinement into hydrogen gas at 85°C (29). This system serves as an example of the key advantages of thermophilic bioprocessing platforms, as it requires minimal sterilization and eliminates energy-intensive cooling of bioreactors, thereby harnessing heat from the associated steel factory for temperature maintenance of the growth vessels (29). CO to hydrogen conversion is accomplished by a native energy-conserving carbon monoxide dehydrogenase (CODH) (30). The *T. onnurineus* CODH was heterologously expressed in *P. furiosus,* where it retained the ATP-generating phenotype enabling CO to serve as an energy source to support robust growth (27, 29, 31). An analogous energy-conserving enzyme of *T. onnurineus,* formate hydrogen lyase (FHL), was also produced in *P. furiosus* for formate-to-hydrogen conversion, but formate oxidation did not support growth (32–34).

A genome-scale metabolic model of *P. furiosus* was recently developed to examine the energy and redox balancing (35). This core model (iGEM_Pfu) includes 624 genes (31.5% of total genes), 647 gene-associated reactions (88.6% of total reactions), and 865 metabolites. The iGEM_Pfu model has been tailored to model metabolically engineered *P. furiosus* strains by adding or removing reactions according to genetic modifications made. These can then be used to identify imbalances or bottlenecks, which may limit product formation (27). A similar model of *Caldicellulosiruptor bescii* was recently used to model heterologous production of 2,3-butanediol, where it was used to identify optimal redox conditions that would increase product yield up to 5-fold the current titer, thus informing future optimization work (36). Herein, *P. furiosus* was engineered to produce 1-propanol and leveraged the metabolic model to identify potential bottlenecks in order to optimize product formation.

## RESULTS AND DISCUSSION

### Strategy for 1-propanol production

The *P. furiosus* strain for 1-propanol production was based on a previously reported strain that produced 3-HP from the disaccharide cellobiose (22). This required three heterologously expressed pathway enzymes (E1–E3) and two accessory enzymes (BL and CA), encoded by a total of seven genes obtained from the archaeon *M. sedula* (T_opt_ 73°C). This facilitates the carboxylation of sugar-derived acetyl-CoA using CO_2_ in an ATP-dependent reaction to 3-HP (Fig. 1). To produce propionyl-CoA from 3-HP, the strategy was to also express in *P. furiosus* the genes encoding three more *M. sedula* enzymes (E4, E5, and E6) (Fig. 1).

Propionyl-CoA can be recognized as a substrate for the native *P. furiosus* enzyme acetyl-CoA synthetase (ACS, PF0973), which generates propionate and conserves energy in the form of ATP (37). Propionate can then be reduced to propionaldehyde by another native *P. furiosus* enzyme, aldehyde ferredoxin oxidoreductase (AOR, PF0346) (21). Finally, to reduce propionaldehyde to 1-propanol, we proposed to utilize the gene encoding primary alcohol dehydrogenase (AdhA) from *Thermoanaerobacter* sp. strain X514 (AdhA_TX514_), a bacterium that grows optimally near 70°C (38, 39). The AdhA enzyme was previously produced in *P. furiosus* for alcohol production to reduce exogenously added propionate to 1-propanol via the so-called AOR/ADH pathway (21).

Hence, the pathway shown in Fig. 1 was expected to generate 1-propanol from cellobiose. The caveat, however, was that the substrate specificities of the final three enzymes in this engineered pathway (ACS, AOR, and AdhA_TX514_) would also allow for the conversion of acetyl-CoA to ethanol, as also shown in Fig. 1. In addition, *P. furiosus* natively produces low concentrations of ethanol from acetaldehyde during fermentation of sugars. This is catalyzed by the Adh encoded by *adhF* (PF0608) in the *P. furiosus* genome, as deletion of *adhF* resulted in the loss of native ethanol production (27). Consequently, if the recombinant strain did indeed generate 1-propanol, we anticipated significant production of ethanol as well, by AdhF*_Pfu_* and/or AdhA*_Tx514_* (Fig. 1).

### Strain construction and verification

*P. furiosus* has a robust genetic system based on an auxotrophic selection system mediated by the gene *pyrF* in the naturally competent Δ*pyrF* strain COM1 (17). This marker can be counter-selected, regenerating the selective phenotype and allowing for multiple rounds of transformation and selection. A strain was generated, which reintroduces *pyrF* into COM1, referred to as COM1C, which is used as a growth and parental control and henceforth referred to as Parent-COM (17). The potential 1-propanol-producing strain, designated PROP (MW670), was generated by the insertion of two synthetic operons into two different genome regions (GRs). We utilized the 3-HP-producing strain (MW130) containing the operon P*_slp_-E1αβγ-E2-E3* driven by the constitutive promoter for the S-layer protein (P*slp*) and located at GR3 (the intergenic region between PF0574 and PF0575) (Table 1). An accessory operon located just upstream contains the biotin protein ligase (BL) and carbonic anhydrase (CA), also from *M. sedula* (22, 25). Strain MW130 was transformed with a linearized plasmid (pHC001) containing a second operon P*_slp_-E4-E5-E6-adhA_TX514_* encoding the three additional *M. sedula* enzymes and the AdhA from *Thermoanaerobacter* sp. strain X514 (Fig. S1). This was inserted into GR1 (the intergenic region between PF0265 and PF0266) with expression driven by P*_slp_*. The ribosome binding sites (RBSs) of heterologously expressed genes *E1α, E1γ, E2*, *E3, E4, E5, E6, and adhA* were replaced with RBSs from well-characterized, high-expression *P. furiosus* genes (Fig. 1). The native *M. sedula* sequence was retained for *E1β*, as *E1α* and *E1β* are transcriptionally coupled (22). All operons include a *P. furiosus* terminator sequence (Fig. 1). The gene encoding the native AdhF was deleted in the PROP strain to determine the contribution of the native AdhF to alcohol production (either 1-propanol or ethanol). A transformable version of the PROP strain (MW675) was generated using 5-FOA counterselection to pop out the *pyrF* cassette. This strain was then transformed with an *adhF* deletion cassette (Fig. S2), generating the PROP-ΔAdhF strain (MW676), referred to here as PROP-ΔAdhF.

**TABLE 1:** Strains used in this study^*a*^

Strain ID	Strain name	Parent	Genotype	Source
	COM1	DSM 3638	Δ*pyrF*	(22)
Parent-COM	COM1C	COM1	Δ*pyrF::* P*_slp_ pyrF*	(17)
	MW076	COM1	Δ*pyrF*::P*_gdh_pyrF* P*_slp_ -E1αβγ-E2-E3*	(22)
Parent-3HP	MW130	MW076	Δ*pyrF*:: P*_slp_ -E1αβγ-E2-E3*	(22)
PROP	MW670	MW130	Δ*pyrF*::P*_gdh_pyrF* P*_slp_ -E1αβγ-E2-E3; P_slp_E4-E5-E6-adhA_TX514_*	This work
	MW675	MW670	Δ*pyrF*:: P*_slp_ -E1αβγ-E2-E3; P_slp_E4-E5-E6-adhA_TX514_*	This work
	MW629	COM1	Δ*pyrF*::P*_gdh_pyrF ; ΔadhF*	(27)
PROP-ΔAdhF	MW676	MW675	Δ*pyrF*::P*_gdh_pyrF* P*_slp_ -E1αβγ-E2-E3; P_slp_E4-E5-E6-AdhA_TX514_ ; ΔadhF*	This work

^*a*^ E1αβγ*,* acetyl/propinyl-CoA carboxylase; E2, malonyl/succinyl-CoA reductase; E3, malonate semialdehyde reductase; E4, 3-hydroxypropionyl-CoA synthetase; E5, 3-hydroxypropionyl-CoA dehydratase; E6, acryloyl-CoA reductase; *adhA_TX514_*, *Thermoanaerobacter* sp. strain X514 primary alcohol dehydrogenase A; *gdh,* glutamate dehydrogenase; P*slp*, *P. furiosus S-*layer gene promoter; *pyrF,* orotidine-5’-phosphate decarboxylase; *adhF*, *P. furiosus* alcohol dehydrogenase F.

### Gene expression of 1-propanol pathway genes

**Fig 2:**
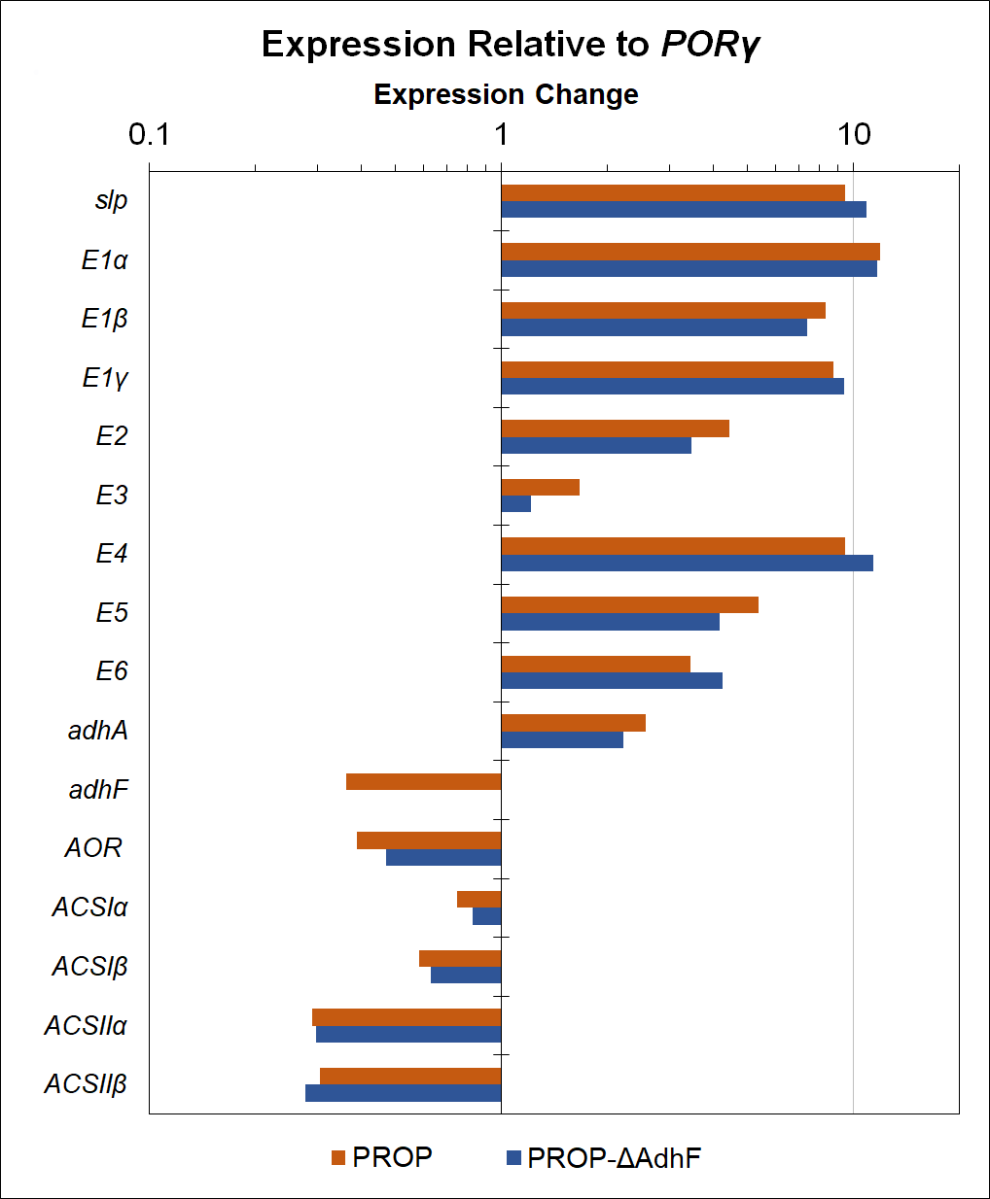
RT-qPCR analysis of pathway genes in 1-propanol-producing strains. PROP (orange) and PROP-ΔAdhF (blue) are shown. Heterologous pathway genes *E1α, E1β, E1γ, E2, E3, E4, E5, E6,* and *adhA_TX514_* are shown, as are native genes *adhF, AOR, ACSIα, ACSIβ, ACSIIα, and ACSIIβ*. Change in gene expression is shown relative to the reference *PORγ*. Technical replicates (*n* = 3) were averaged for each strain.

The heterologous genes *E1α, E1β, E1γ, E2, E3, E4, E5, E6,* and *AdhA*_TX514_ are under control of the high-copy P*_slp_*. qPCR was performed to verify transcription of these heterologous genes, determine their relative expression, and verify the deletion of *adhF* in the PROP-ΔAdhF strain. The gene expression of native genes in the 1-propanol pathway was also evaluated, including *AOR, ACSIα, ACSIβ, ACSIIα,* and *ACSIIβ*. This analysis used expression of the native *P. furiosus* pyruvate ferredoxin oxidoreductase (POR) subunit γ (*PORγ,* PF0971) gene as a reference, with the gene encoding the S-layer protein (*slp,* PF1399) included as an additional control. The heterologous genes expressed previously for 3-HP production (*E1α, E1β, E1γ, E2,* and *E3*) are highly expressed in both of the 1-propanol strains, indicating that overexpression of the operon was maintained in these new strains. The four newly inserted genes under P*_slp_* control (*E4, E5, E6,* and *adhA*) also show increased expression relative to *PORγ,* showing efficient high copy expression of this new operon in both PROP and PROP-ΔAdhF (Fig. 2). The level of *adhF* expression in PROP is consistent with previous work, whereas in PROP-ΔAdhF expression is below the level of detection, indicating the locus has been effectively deleted (27, 37, 40). The expression levels of the AOR and ACS genes are consistent between PROP and PROP-ΔAdhF and are consistent with previous work with *P. furiosus* (41).

### 1-Propanol production

**Fig 3:**
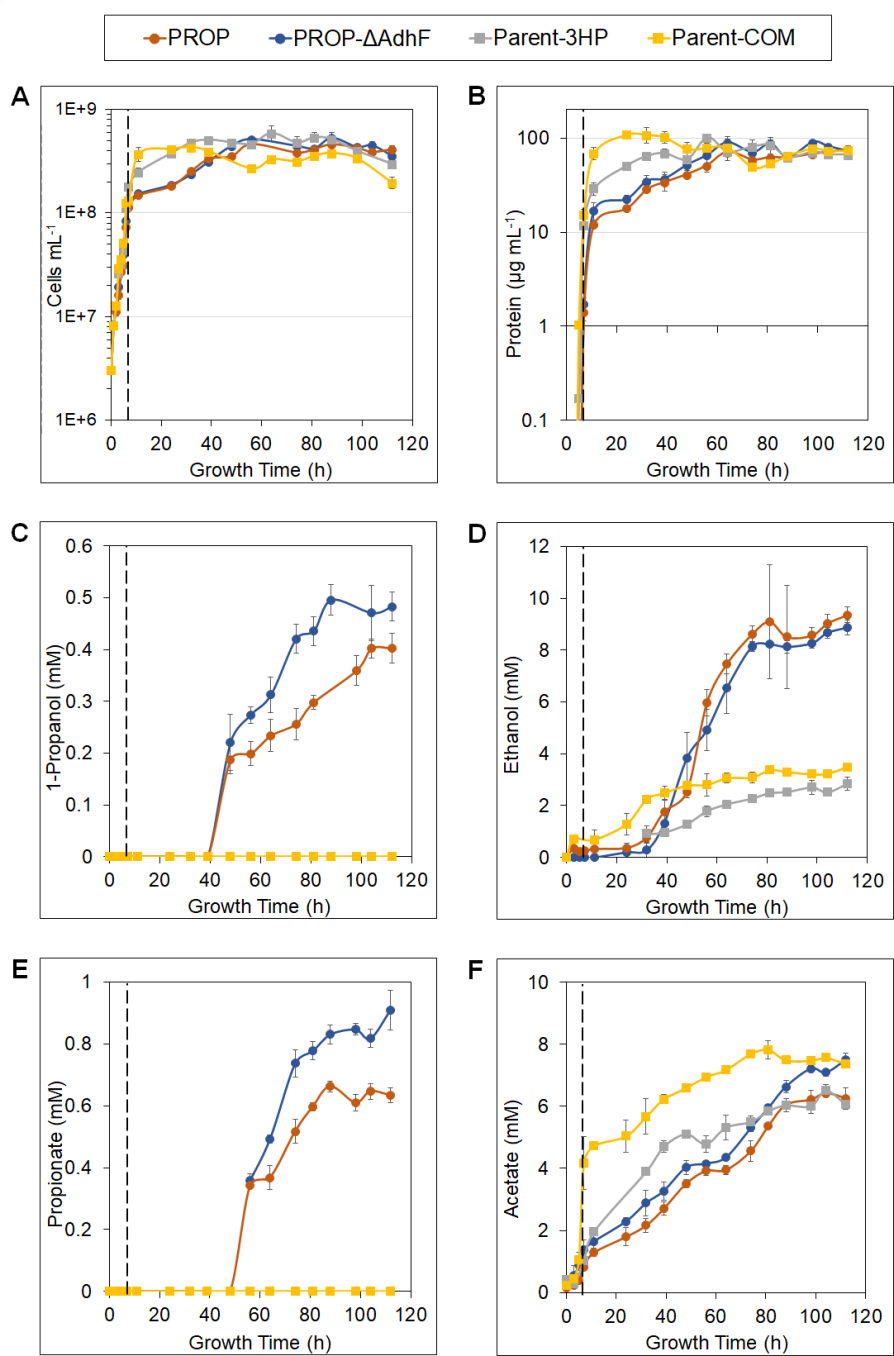
Temperature shift growth (98°C-75°C) of 1-propanol and parent strains. Strains PROP (orange circles), PROP-ΔAdhF (blue circles), Parent-3HP (gray squares) and Parent-COM (yellow squares) are shown. A growth temperature of 98°C was maintained until cell density reached 1 × 10^8^ cells/mL (hour 7, dashed line). The temperature was then maintained at 75°C. Error bars represent standard error; *n* = 4 for each strain. (A) Cell density in cells/mL of culture; (B) protein concentration in μg/mL; (C) 1-propanol, (D) ethanol, (E) propionate, and (F) acetate concentration in mM present in spent media.

Due to the lower growth temperatures of both *M. sedula* (T_opt_ 73°C) and *T*. strain X514 (T_opt_ 70°C) compared with *P. furiosus* (T_opt_ 100°C), a temperature shift approach to growth was utilized as previously described (Fig. 3A and B) (22). The recombinant *P. furiosus* strains PROP and PROP-ΔAdhF were not expected to produce 1-propanol from cellobiose at 98°C due to inactivation of the heterologously expressed enzymes, and this proved to be the case (Fig. 3C). The recombinant *P. furiosus* strains were grown at 98°C until a density of approximately 1 × 10^8^ cells/mL was reached, corresponding to mid-late exponential phase (Fig. 3A), after which time (7 h) cultures were placed at 75°C and incubated for an additional 105 h. Under these conditions, 1-propanol was produced by both strains with endpoint concentrations of 0.40 ± 0.02 mM and 0.49 ± 0.02 mM for PROP and PROP-ΔAdhF, respectively (Fig. 3C). Neither the parent strain of PROP (Parent-3HP) nor its parent (Parent-COM) produced any detectable 1-propanol (or propionate, Fig. 3). Wild-type *P. furiosus* has a doubling time of approximately 5 h at 75°C (16) compared with 40 min or so near the temperature optimum (15). Hence, by employing the temperature shift strategy, growth time was minimized without sacrificing 1-propanol production (Fig. 3; Fig. S3).

**Fig 4:**
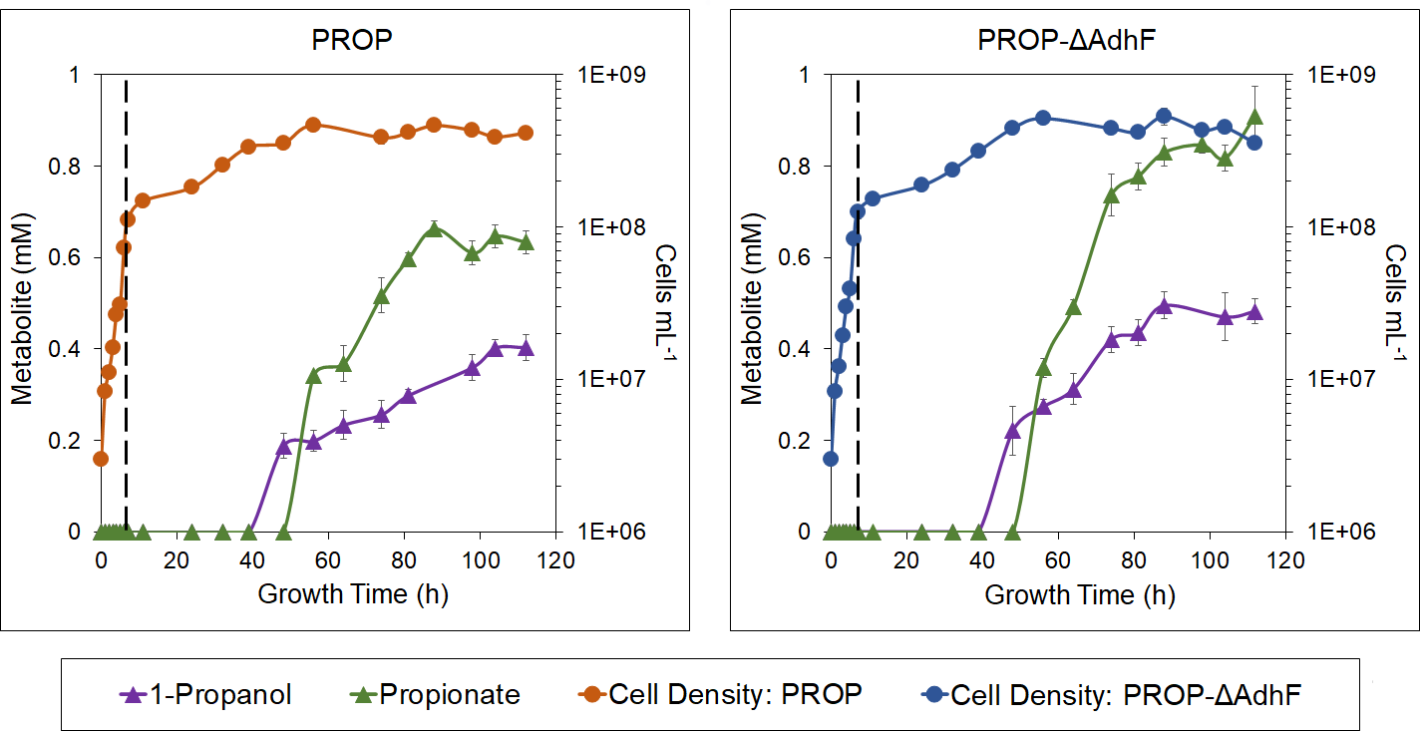
Production of 1-propanol and propionate versus cell density. 1-propanol (purple triangles) and propionate (green triangles) concentrations are shown (left axis) alongside cell density (blue and orange circles, right axis). Error bars represent standard error, *n* = 4.

The 1-propanol pathway intermediate propionate is also produced by PROP and PROP-ΔAdhF, with initial detection at time point 56 h in both strains. Endpoint concentrations of propionate were 0.63 ± 0.03 mM in PROP and 0.90 ± 0.06 mM in PROP-ΔAdhF, indicating an accumulation of this 1-propanol precursor (Fig. 3). This suggested that although propionate production may be driven by the ATP generated by ACS, the last two steps of the pathway, AOR and AdhA, are limiting. However, the presence of propionate in spent media further shows the functionality of the *M. sedula* E1–E6 portion of this pathway. The relationship between the growth phase, cell density, and production of 1-propanol and propionate are shown in Fig. 4. Both products are initially detected at or near the maximum cell density of the cultures, continuing to accumulate as the cultures remain in the stationary phase. These results suggested that the production of 1-propanol and propionate is not taking place at a significant rate until the cultures are stationary. This trend is present in both PROP and PROP-ΔAdhF. It is also of note that propionate in its protonated form is able to diffuse across the membrane. For example, it was previously shown that 1-propanol was produced when propionate was added exogenously to an engineered AOR/ADH (21). Therefore, it is possible that some propionate found in the medium may re-enter the cell and be reduced to 1-propanol by the final two steps of the pathway, although it is not possible to quantitate this.

Acetate is the main end product of *P. furiosus* fermentation, but it is also the precursor to ethanol, a major off-target product in this engineered system (Fig. 1). Acetate production was not significantly different between the two PROP strains, with endpoint values of 6.26 ± 0.33 mM and 7.49 ± 0.23 mM in PROP and PROP-ΔAdhF, respectively (Fig. 3F). Acetate did not differ significantly between the parent strains, with endpoint values of 6.07 ± 0.15 mM in Parent-3HP and 7.37 ± 0.13 in Parent-COM. Due to the AdhA_TX514_ being expressed in PROP and PROP-ΔAdhF, ethanol production was higher than in Parent-3HP and Parent-COM, consistent with previous work (21). The endpoint concentration of ethanol was 9.36 ± 0.31 mM in PROP and 8.98 ± 0.30 mM in PROP-ΔAdhF, whereas the parental controls were 2.85 ± 0.26 mM in Parent-3HP and 3.49 ± 0.06 mM in Parent-COM. In terms of growth, note that after the temperature is shifted from 98°C to 75°C at time point 7 h, the 1-propanol-producing strains experience another lag in growth, which is not seen in the Parent-3HP and Parent-COM strains (Fig. 3A). Although the peak density for these parent strains is reached at time points 32 h and 24 h, respectively, peak cell density for PROP and PROP-ΔAdhF is reached at 56 h of growth. This trend is reflected in the protein concentration for these strains as well, with the 1-propanol strains reaching maximum protein concentration at 64 h of growth.

To determine if the *P. furiosus* strains could produce 1-propanol while actively growing and whether this would affect the distribution of the other products, PROP, PROP-ΔAdhF, and the two parent strains were grown at 75°C with no temperature shifting. Although 1-propanol was detected in the culture medium of both PROP strains but not in the parent strains, this did not occur until well into the stationary phase (after approximately 100 h; see Fig. S3). The 1-propanol concentrations were 0.42 mM in PROP and 0.48 mM in PROP-ΔAdhF after approximately 180 h, with similar concentrations of propionate. These concentrations are virtually identical to those obtained with the temperature shift approach, but it took nearly twice as long. Deletion of the native AdhF had the effect of significantly increasing the rate of production of both 1-propanol and propionate, although the final concentrations were comparable. However, both PROP and PROP-ΔAdhF exhibited growth phenotypes compared with the parent strains: extended lag and exponential growth phases with cultures not reaching maximum cell density until 107 h of growth (Fig. S3A and B). In contrast, Parent-COM and Parent-3HP reached the maximum cell density at 69 h. As was seen in the temperature shift growth, the PROP strains produce significantly more ethanol than the parent strains (Fig. 3D; Fig. S3D). Endpoint concentrations of ethanol in PROP and PROP-ΔAdhF were 8.19 ± 0.42 mM and 8.9 ± 0.07 mM, respectively, compared with 9.39 mM and 8.98 mM in the temperature shift approach. The control strains Parent-3HP and Parent-COM, which lack the heterologous AdhA_TX514_, were 1.56 ± 0.36 mM and 2.30 ± 0.67 mM, respectively. This is slightly lower than the temperature shift growth, which measured 2.85 mM and 3.49 mM.

### Carbon balancing of 1-propanol-producing strains

**Fig 5:**
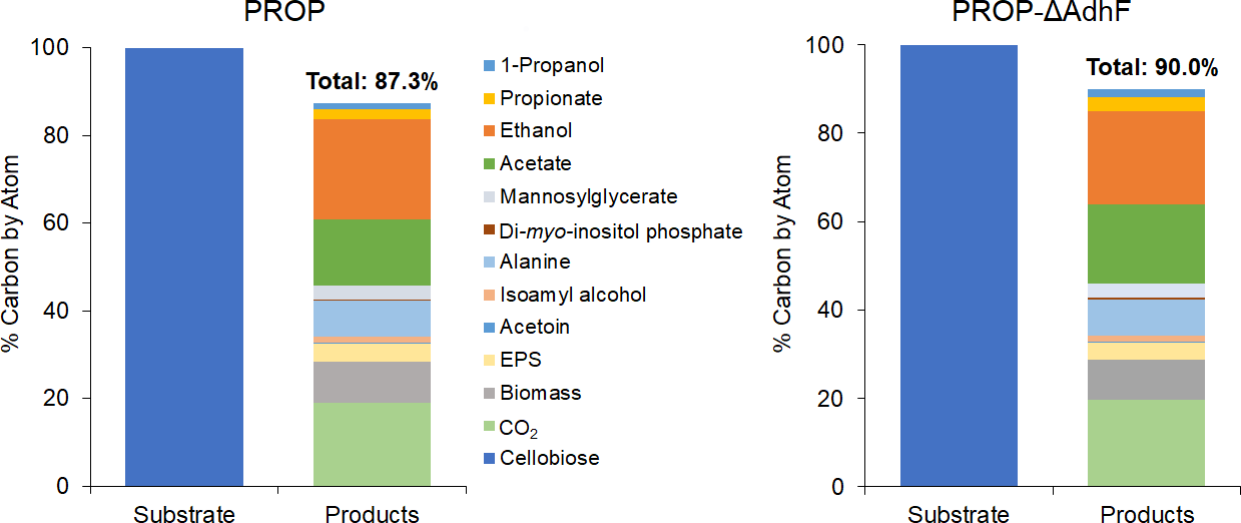
Carbon balances for strains PROP and PROP-ΔAdhF from temperature shift growth (98°C–75°C). Values shown represent percent carbon by atom, with the substrate (cellobiose) utilized being set to 100%. Products 1-propanol, propionate, ethanol, acetate, isoamyl alcohol, and acetoin were measured with endpoint concentration used in calculations. Values for CO_2_, mannosylglycerate, di-*myo*-inositol phosphate, alanine, EPS, and biomass were calculated from growth and metabolite data.

In order to track carbon consumption and its relationship to 1-propanol and other product formation, carbon distribution was determined after the temperature shift growth of PROP and PROP-ΔAdhF (Fig. 3). The starting concentration of cellobiose was 10.3 mM for both strains. Strain PROP and PROP-ΔAdhF consumed 6.8 ± 0.53 mM (*n* = 3) and 7.0 ± 0.24 mM (*n* = 3), respectively, and these were used as the 100% values in calculations (Fig. 5). In PROP, 87.3% of carbon consumed in the form of cellobiose was accounted for in products, whereas 90.0% of carbon was accounted for in PROP-ΔAdhF (Fig. 5). The target product 1-propanol accounted for 1.4% of total carbon in PROP and 1.7% in PROP-ΔAdhF, whereas propionate accounted for 2.3% and 3.3%, respectively. The off-target product ethanol accounted for 22.7% of PROP and 21.1% of PROP-ΔAdhF, with its corresponding acid acetate accounting for 15.2% and 17.9%, respectively. For carbon closure purposes, CO_2_ output was calculated based on metabolite data (see Materials and Methods). PROP and PROP-ΔAdhF had similar calculated CO_2_ contributions, at 19.1% and 19.6% carbon recovered, respectively. Ultimately, the distribution of carbon recovered did not differ significantly between these two strains. The similarity of the 1-propanol and ethanol values between strains also indicates that the native AdhF is either not playing a significant role in PROP or, in the absence of AdhF, the heterologous AdhA*_TX514_* was able to accommodate the flux through each of these pathways. Although around 90% of carbon is recovered in the 1-propanol-producing strains, the potential for other minor products resulting from the 1-propanol pathway cannot be ruled out, particularly when considering the relative promiscuity of enzymes such as the native AOR and heterologous AdhA*_TX514_*. These could include 1,3-propanediol derived from 3-hydroxypropionate. However, we were unable to detect this potential product in the spent media.

### 1-Propanol production and cellobiose consumption

**Fig 6:**
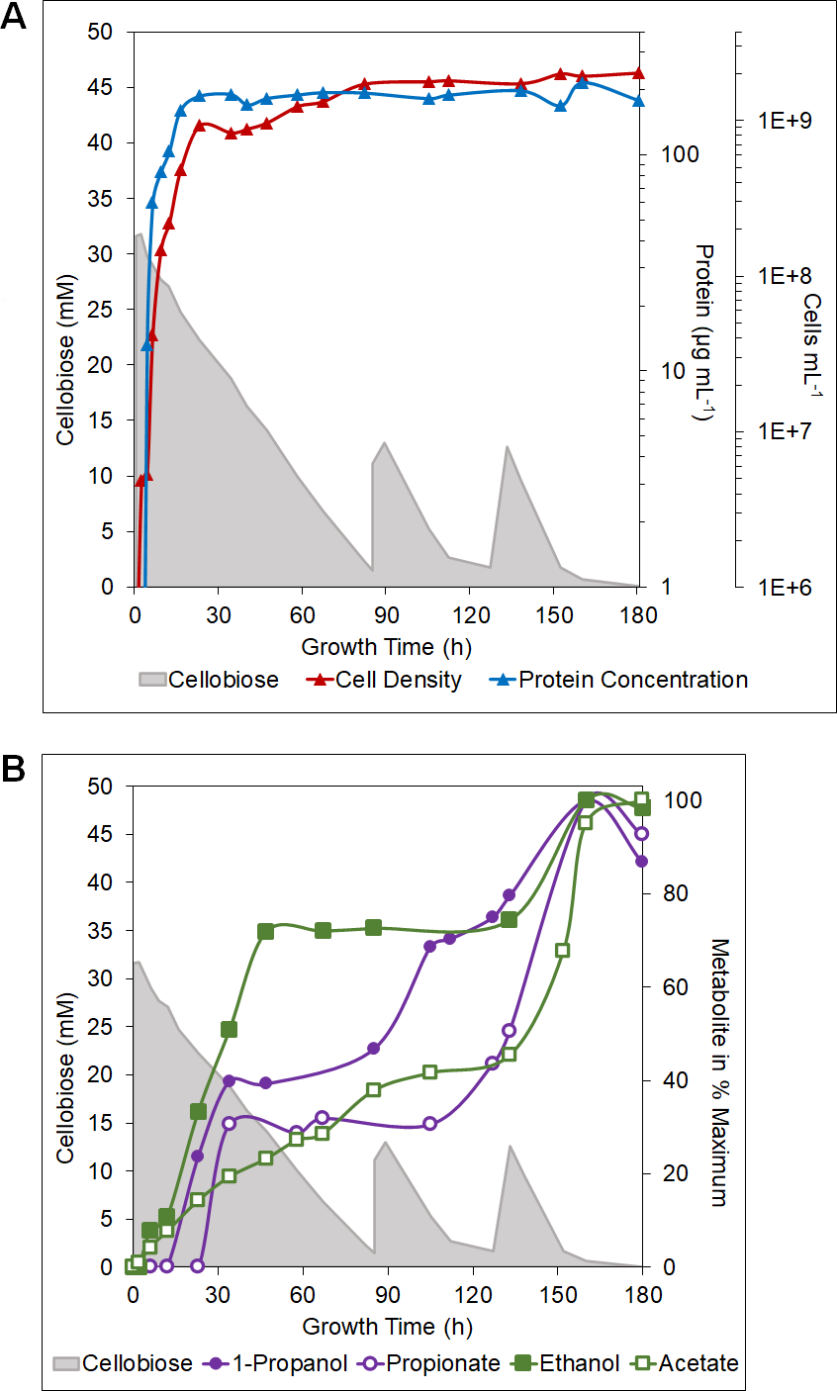
Effect of additions of cellobiose on 1-propanol production by strain PROP. Strain PROP was grown at 98°C until cell density reached 1 × 10^8^ cells/mL (hour 6) then maintained at 75°C. (A) Cellobiose concentration with respect to growth. Cellobiose concentration in media (gray area, left axis), protein concentration in μg/mL (blue triangles, right axis, left scale), and cell density in cells/mL of culture (red triangles, right axis, right scale). (B) Metabolite production with respect to cellobiose concentration. Metabolites are represented in % maximum (right axis), 1-propanol (purple circles, closed), propionate (purple circles, open), ethanol (green squares, closed), and acetate (green squares, open).

As noted above, the temperature shift cultures consumed approximately 60% of the initial cellobiose (10 mM) and converted about 3% of its carbon to equal concentrations of 1-propanol and propionate (about 1 mM total). To determine how product distribution was related to cellobiose consumption, we switched to a stirred, pH-controlled fermentation system. This approach can also increase final cell densities and product generation compared with pH-uncontrolled static cultures (27). For example, this approach increased ethanol production by approximately 4-fold using a strain in which the native AdhF was overexpressed (27). The PROP strain was grown in a 15 L fermenter using 30 mM cellobiose, and the medium pH was held at pH 6.0 by the addition of sodium bicarbonate. Once the culture reached the target 1 × 10^8^ cells/mL (after 6 h), the temperature was lowered to 75°C. As anticipated, much higher cell densities were obtained (2 × 10^9^ cells/mL; Fig. 6A) compared with growth in static bottles (4 × 10^8^ cells/mL; Fig. 3A). This was also reflected in protein concentration, which remained near 200 µg/mL during the stationary phase (Fig. 6B).

In the fermenter with cells growing on 30 mM cellobiose, 1-propanol was detected earlier than in bottles, with 0.22 mM produced at 23 h, but this leveled off at 35 h, although cells continued to produce acetate and use cellobiose (Fig. 6). This was exhausted at 85 h and upon adding a further 10 mM cellobiose, 1-propanol production resumed, reaching approximately 0.7 mM at 120 h, when the cellobiose had been used up. Further addition of cellobiose (10 mM) led to resumption of 1-propanol production, and the maximum (0.95 mM) was reached at 160 h, when once more cellobiose was exhausted. Hence, 1-propanol production was directly dependent upon cellobiose utilization.

This was not true with propionate or ethanol, the concentrations of which did not change upon addition of the first 10 mM cellobiose but did to the second 10 mM, with the final concentrations of propionate and 1-propanol being comparable (Fig. 6). However, a total of 50 mM cellobiose was consumed to generate approximately 2 mM of 1-propanol and propionate, which is almost an order of magnitude lower than the yields of these compounds seen in bottles (6 mM cellobiose yielded almost 1 mM total 1-propanol and propionate). There was also a major difference seen in final ethanol yield, where the concentration in the fermenter (7.5 mM from 50 mM cellobiose) was similar to that measured in the bottles (8.5 mM from 6 mM cellobiose). However, almost 10-fold more cellobiose was consumed. In contrast, the final acetate yields, 40 mM acetate from 50 mM cellobiose in the fermenter and 6 mM from 6 mM cellobiose in bottles were essentially the same in both modes of culturing the PROP strain. This is consistent with cell growth being directly related to acetate production, independent of the growth conditions. Clearly, the direction of carbon flux to 1-propanol, propionate, and ethanol was very dependent upon the growth conditions, and how they can be changed to optimize 1-propanol production is not clear. A metabolic modeling approach was, therefore, considered a potential means of increasing yields of the desired product.

### Identifying metabolic bottlenecks for 1-propanol production

Genome-scale metabolic modeling of the PROP strain predicted the theoretical potential of 1-propanol production and helped evaluate the strategies for increasing 1-propanol yield. The model was constructed by inserting the engineered 1-propanol-producing pathway into a previous publication of the *P. furiosus* metabolic model (35). With exchange constraints calibrated based on the media conditions and measurements of cellobiose consumption (6.87 mM) and protein yields (93.65 µg/mL) in stationary, pH uncontrolled cultures of PROP. The model predicted a maximum production of up to 5.9 mM 1-propanol, much greater than the experimentally measured 1-propanol yield of 0.40 mM (Fig. 3C). This indicates that the current PROP strain is far from reaching its theoretical potential, although the model may not fully represent the potential constraints that limit 1-propanol production.

**Fig 7:**
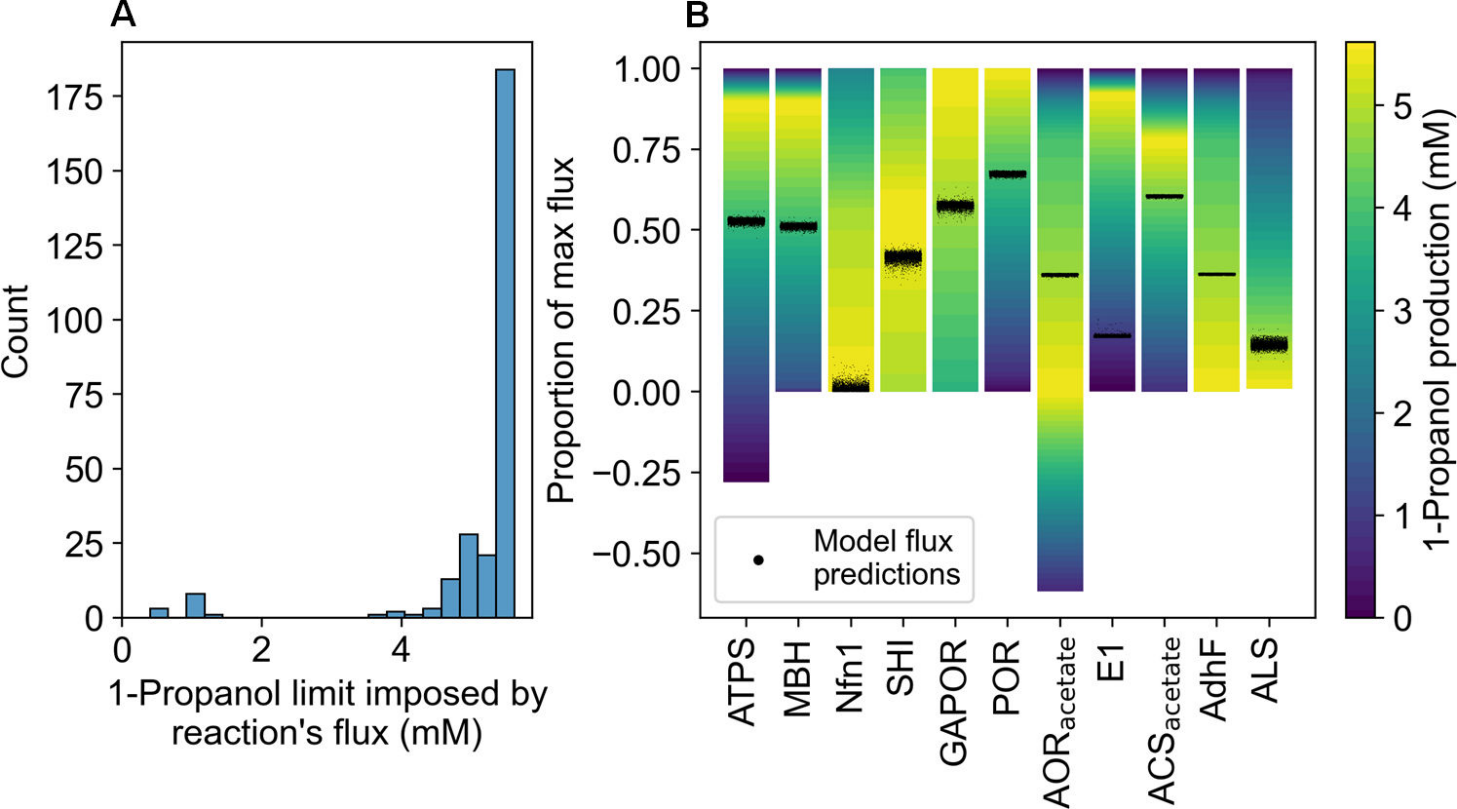
Analysis of reaction fluxes in PROP. (A) Histogram showing the distribution of maximum 1-propanol flux when individual metabolic reactions were constrained based on the medium fluxes from random sampling, using conditions in bottled cultures of the PROP strain. (B) Model-predicted fluxes for the PROP cultures (black points) compared with flux ranges of individual reactions under increasing 1-propanol yield (colored gradient). The flux of each reaction was scaled to the range [−1, 1], where positive numbers represent flux in the forward direction and negative numbers represent flux in the reverse direction. Most enzymes had predicted fluxes within or near their optimal ranges for 1-propanol production (green-yellow regions), except for E1, which has fluxes far lower than its optimal range (blue region).

In order to identify potential bottlenecks, we calculated the maximum 1-propanol yield while limiting the flux for each metabolic reaction to the median value observed from random flux sampling under physiological conditions of the bottled PROP culture. The results showed a multimodal distribution, with most reactions compatible with a 1-propanol yield of 4–6 mM, indicating that these reactions are unlikely to be primary bottlenecks (Fig. 7A). Twelve reactions were flagged as possible bottlenecks, as their median flux values from metabolic flux sampling limited the maximum 1-propanol yields to less than 2 mM. The 12 bottleneck reactions included the entire 1-propanol pathway starting from acetyl-CoA, including E1-E6, ACS, AOR, AdhA, and 1-propanol export (Fig. 1), inorganic pyrophosphatase (EC 3.6.1.1), and the non-enzymatic conversion of CO_2_ to bicarbonate (Data S1). The latter two reactions are highly correlated with the 1-propanol pathway because the pathway consumes CO_2_ and generates pyrophosphate. This suggests that selectivity toward the 1-propanol pathway is the primary limitation for 1-propanol production in the existing PROP strain.

To search for engineering designs that enhance the selectivity for 1-propanol production, we compared the measured flux distributions to the theoretical flux ranges under increasing 1-propanol yields for 11 central metabolic reactions (Fig. 7B). These included four key redox and energy metabolism reactions: ATP synthase (ATPS), membrane-bound hydrogenase (MBH), NADH-dependent ferredoxin NADP^+^ oxidoreductase (Nfn1), and NADP^+^-dependent soluble hydrogenase I (SHI); three redox reactions in glycolysis and product fermentation pathways: GAP ferredoxin oxidoreductase (GAPOR), pyruvate ferredoxin oxidoreductase (POR), and aldehyde ferredoxin oxidoreductase (AOR_acetate_); and four reactions that are committed steps in product pathways branching from pyruvate: E1, acetyl-CoA synthetase (ACS_acetate_ – acetate production), alcohol dehydrogenase (AdhF – ethanol production), and acetolactate synthase (ALS – acetoin and branched-chain amino acid synthesis). The distribution of E1 fluxes fell far below their target range, demonstrating that selectivity toward the 1-propanol pathway must be improved to maximize 1-propanol production. Additionally, the fluxes of acetate synthesis (ACS_acetate_ flux) and ATP production (ATPS and MBH fluxes) were lower than their target values for 1-propanol optimization. Note that the MBH flux is also included in the ATP calculation as this enzyme evolves H_2_ and pumps Na^+^ ions, which are used by ATPS to generate ATP. The model suggested that increasing the flux of POR while reducing the flux of AdhF and ALS would benefit 1-propanol production by channeling as much pyruvate as possible to acetate and 1-propanol pathways. Nfn1 and SHI were in their optimal ranges, suggesting that no modification is needed for these enzymes.

Based on these results, we hypothesized that 1-propanol production in the PROP strain was limited by the availability of ATP. We tested this hypothesis by introducing artificial reactions that freely generate ATP from ADP. Additionally, we examined if reductant was a limiting factor by introducing a reaction that freely generates NADPH from NADP^+^. We observed that increasing the amount of ATP directly increased the theoretical 1-propanol yields, whereas increasing the amount of NADPH had no effect (Fig. S6). Therefore, new engineering designs of the PROP strain should seek to alleviate limitations in ATP production.

### Conclusions

This work establishes a hybrid metabolic pathway in *P. furiosus* for the production of 1-propanol from carbohydrate-derived acetyl-CoA. This nine-step pathway consists of six enzymes from the 3-HP/4-HB cycle of the thermophilic archaeon *M. sedula*, the primary alcohol dehydrogenase A from thermophilic bacterium *Thermoanaerobacter* sp. strain X514, and two native enzymes from *P. furiosus*. This work establishes a new engineered bioproduct for this organism, joining lactate, butanol, 3-hydroxypropionate, and ethanol (19–22, 27, 28). The recombinant strains of *P. furiosus* described herein produce the target 1-propanol at 75°C, 25°C below the organism’s optimum growth temperature, through both sub-optimal growth and temperature-shift approaches. This represents a very high temperature for the biological production of 1-propanol, thus potentially taking advantage of the key benefits associated with high-temperature fermentations (14). The highest 1-propanol yield described here is 0.95 mM, which was achieved in a sparged and pH-controlled fermenter. The genome-scale metabolic model of *P. furiosus* was harnessed to identify bottlenecks in carbon and electron flow to 1-propanol and potential optimization targets in this system. Simulations identify ATP as a significant linting factor in 1-propanol production and suggest that improving selectivity toward the 1-propanol pathway is necessary. Solutions to these issues can be explored experimentally in future optimization efforts.

## MATERIALS AND METHODS

### Construction of plasmids and PCR products

The 1-propanol pathway was constructed by taking advantage of an existing *P. furiosus* strain, MW130, that generates 3-HP (22). The plasmid (pGL006) in this strain contains the following genes in an operon behind a P*_slp_* promoter: *E4* (Msed_1456), *E5* (Msed_2001), and *E6* (Msed_1426) as well as a pSC101 origin and a copy of the P*_gdh_-pyrF* (*gdh*: PF1602; *pyrF*: PF1114) uracil prototrophic selection marker system (17, 22). Homologous upstream and downstream regions flank the operon on pGL006 for incorporation into the *P. furiosus* PF0265–PF0266 intergenic region (designated GR1), a site previously identified to have very little to no transcriptional activity (18). A PCR-based approach was used to introduce *adhA* (TX514_0564) to the 5’ end of the existing operon. The *adhA* coding sequence was amplified from *Thermoanaerobacter* sp. strain X514 genomic DNA, whereas pGL006 was amplified from purified plasmid product isolated from *E. coli*. Both PCR reactions were designed with overhanging regions of homology to orient the two fragments. The NEBuilder seamless cloning kit (New England Biolabs) was used for ligation, completing both the synthetic operon and *E. coli* insertion vector. The construct was transformed into 10-β competent *E. coli* cells (NEB), with resulting colonies being screened and plasmid DNA sequence verified. The resulting plasmid is termed pHC001 and contains the newly constructed P*_slp_-E4-E5-E6-adhA_TX514_* operon as well as the features described above from pGL006 (Fig. S1). A construct for the deletion of the *P. furiosus adhF* gene was previously constructed through overlap PCR (27). This construct contains the P*gdh-pyrF* marker flanked by 500 bp of homology flanking the chromosomal AdhF gene. For this work, the resulting deletion strain (MW629) was revived from glycerol stock. Genomic DNA was extracted using the Quick-DNA microprep kit (Zymo Research) and the construct was amplified using PCR. Primer sequences for this work can be found in Table S1 in the supplemental materials.

### Strain construction

Transformation was performed as previously described for *P. furiosus,* with linearized plasmids and purified PCR products being used as donor DNA (17). To generate the initial 1-propanol strain (MW670, PROP), pHC001 was linearized via digestion with *NotI* restriction enzyme. The construct was transformed into a previously developed strain MW130 (P*_slp_ -E1αβγ-E2-E3*) (22). For further engineering of PROP, 5-fluoroorotic acid (5-FOA) was used as previously described (17, 22) to select for the loss of the P*_gdh_-pyrF* marker construct. This generated a transformable version of the PROP (MW675), which lacks *pyrF* and the ability to grow without exogenous uracil. To generate a 1-propanol-producing strain harboring a native *adhF* deletion, MW675 was transformed with the *adhF* deletion construct to generate PROP-ΔAdhF (MW676) (*E4-E5-E6-adhA_TX514_;ΔadhF*). This deletion construct was obtained via PCR using a previously developed *adhF* deletion strain (MW629 [27]). Regions of insertion in each strain and the *adhF* deletion in PROP-ΔAdhF were verified through Sanger Sequencing. Strains utilized and constructed in this work are detailed in Table 1.

For qPCR analysis of recombinant gene expression, 300 mL of cultures were grown to mid-log phase at 75°C and aliquoted into 50 mL Falcon tubes, and the cells were pelleted at 5,500 rpm for 15 min at 12°C. The supernatant was decanted, and the pellets were immediately flash-frozen in a bath of isopropanol and dry ice. Pellets were stored at −80°C until RNA extraction. RNA was isolated by first performing a phenol:chloroform (5:1) extraction as previously described (17, 22). Initial extraction was treated using TURBO DNase1 (Invitrogen) for the digestion of genomic DNA. Final purification of RNA was then performed using the Direct-zol RNA miniprep kit (Zymo Research). For use in qPCR, cDNA was synthesized from purified RNA using the Affinity Script qPCR cDNA synthesis kit (Agilent). For qPCR analysis, Brilliant III Ultra-Fast SYBR green qRT-PCR master mix was used (Agilent), and reactions were performed on an Mx3000P instrument (Stratagene). The gene encoding the gamma subunit of the *P. furiosus POR* (PF0971) was included as a reference gene and internal control.

### Growth of *P. furiosus*

For genetic manipulation, *P. furiosus* strains were grown as previously described (17) in a defined seawater-replicating medium containing 3.5 g/L (10.22 mM) cellobiose as the carbon source unless stated otherwise. The pH values of the media were adjusted to 6.8 with NaOH and HCl when needed. For strains lacking *pyrF,* media were supplemented with 20 µM uracil. For growth experiments, minimal media was used as described above but supplemented with 0.5 g/L yeast extract. Growth experiments for 1-propanol production were performed at 75°C or by a temperature shift approach where cultures were grown at 98°C until mid-log phase (cell density 1 × 10^8^ cells/mL), and the temperature was then adjusted to 75°C. Transformations were performed on plate media as previously described (17).

For product analyses, *P. furiosus* strain PROP was grown in a 20 L stainless-steel pH and temperature-controlled fermenter, with a culture volume of 15 L (Fig. 6). Media composition was as previously described (17), with exceptions: 0.5 g/L yeast extract was added, pH was adjusted to 6.0 with NaOH and HCl, and a starting concentration of 30 mM cellobiose was established. The fermenter was agitated at 75 rpm, sparged with N_2_CO_2_, and held at pH 6.0 using sodium bicarbonate titrated through an automatic baseline. Cellobiose concentration was monitored throughout growth, with the culture being fed an additional 10 mM cellobiose when concentration neared zero (Fig. 6)

### Metabolite and cellular protein analysis

During growth curves, the culture was collected at regular intervals, which varied based on growth temperature and doubling times (8–12 h for growth at 75°C and 1–2 h for growth above 85°C); 1 mL of the sample was harvested from culture bottles using a syringe and deposited in a 96-well deep-well plate. After allowing the samples to cool to room temperature, the plates were centrifuged at 4,100 rpm for 30 min at 10°C. Spent media was pipetted into new 96-well deep-well plates and sealed with plate sealing foil prior to being stored at −20°C. The remaining medium was decanted, and the cell pellets were used for protein quantitation using a standard Bradford protein assay (Bio-Rad) as described previously (17). Cell density was determined via cell counts performed using 2 µL fresh culture and counted using a Petroff-Hauser cell counting chamber. For metabolite analysis, 100 µL spent media were acidified with 5 µL of 2 M phosphoric acid. Acidified samples were analyzed by GC-FID (Agilent 7890A; columns: Restek Stabilwax 30 m × 0.25 mmID ×0.25 µm and Agilent DB-Wax UI 30 m × 0.32 mm × 0.25 µm). Standard curves of key compounds were used for metabolite quantitation, including 1-propanol, ethanol, acetate, and propionate. For the quantification of cellobiose in spent media, 90 µL of the sample was acidified with 10 µL 1 M H_2_SO_4_. Samples were measured using HPLC-RID (Aminex 87H column with 5 mM H_2_SO_4_ as eluent). A 6-point standard curve ranging from 0.5 mM to 15 mM cellobiose was used for quantification.

Metabolite, growth, and carbon utilization data were used to perform carbon balancing in strains PROP and PROP-ΔAdhF. This was performed per atom, represented in mM carbon consumed and recovered (Fig. 5). The substrate input is cellobiose consumed, which was set to 100% for product calculations. 1-propanol, propionate, ethanol, acetate, acetoin, and isoamyl alcohol were measured using GC-FID as described above. *P. furiosus* POR is responsible for the oxidative decarboxylation of pyruvate, reducing ferredoxin and yielding acetyl-CoA and CO_2_ (42). For carbon balancing, the CO_2_ produced from ethanol, acetate, and acetoin (43) was accounted for using the following equation:


$$mM\;acetate+mM\;ethanol+\left(mM\;acetoin\times 2\right)=mM\;CO_{2}$$

Although the 1-propanol pathway does rely on native acetyl-CoA production, the first step in the heterologous pathway, E1, is CO_2_ utilizing (Fig. 1). Therefore, the production of 1-propanol is CO_2_-neutral. The concentrations of di-*myo*-inositol phosphate and mannosylglycerate, two compatible solutes associated with osmotic and temperature stress tolerance in *P. furiosus,* were calculated using measured protein concentrations (44), and alanine production was estimated based on cellobiose consumed (44, 45). Exopolysaccharide (EPS) was purified and measured as previously described (46).

Hydrogen production was measured by sampling the headspace of sealed 50 mL cultures using a Pressure-Lok syringe (Vici Precision Sampling). The samples were measured as previously described (32) using a Shimadzu Gas Chromatograph GC-8A (Restek MoleSieve 5A 80/100 column, 6 ft 2 mm).

### Genome-wide metabolic modeling

The genome-scale metabolic model of *P. furiosus* (35) was simulated using PSAMM version 1.2.1 (for flux balance analysis and flux variability analysis) and cobrapy version 0.26.2 (for random flux sampling) packages in Python 3.9.15 (47, 48). All simulations that reference closed culture conditions use the default GEM-iPfu model. Simulations referencing open culture conditions have the reaction fluxes of the two cytoplasmic hydrogenases (SHI and SHII) constrained to zero (R07181 and R00700, respectively), following conventions established in our previous study (35). For all simulations, the exchange reactions were formulated to match the media used for bottle cultures that contained 3.5 g/L cellobiose as the carbon source and 0.5 g/L yeast extract. Protein yield, cellobiose consumption, and production of compounds measured in the carbon closure analysis were limited to match experimentally measured values from the PROP cultures after 112 h of growth.

Flux sampling was performed using the cobrapy sample function, which implements the OptGpSampler method (49). To estimate the distribution of reaction fluxes for the PROP strain (Fig. 7), exchange limits were set based on experimental measurements (see above). The feasible flux ranges of metabolic reactions were calculated using the fva function in PSAMM, with 1-propanol constrained in 40 steps of increasing values between 0 and 5.6 mM, where the latter value represents the theoretical maximum predicted by the model.

Flux balance analysis was used to simulate the addition of unlimited or free sources of ATP or NADPH (Fig. S6). Since the model is a steady-state model, metabolites cannot be directly added as this would violate the assumption of constant metabolite concentration. Instead, we added balanced reactions that generate each of the desired energy or redox compounds, and we performed simulations by varying the flux of these reactions and observing their effects on 1-propanol production. The reactions were defined as follows:


$$\begin{array}{ll} Free\;ATP\;:\;P_{i}+ADP\;\to \;ATP+H_{2}O & \\ Free\;NADPH\;:\;NADP^{+}+H_{2}O\;\to \;NADPH+H^{+} \end{array}$$

## Data Availability

The *P. furiosus* genome-scale model, GEM-iPfu, is available on GitHub at the following address: https://github.com/zhanglab/GEM-iPfu. YAML and SBML formatted models, including all inputs and analysis scripts used in this study, are released at the following address: https://doi.org/10.5281/zenodo.15008570.

## SUPPLEMENTAL MATERIAL

Data File S1 aem.00471-25-s0001.xlsx Maximum propanol yields predicted by the PROP model when individual reactions were constrained based on the median of randomly sampled flux distributions.

Supplemental material aem.00471-25-s0002.pdf Table S1 and Figures S1 to S6.

ASM does not own the copyrights to Supplemental Material that may be linked to, or accessed through, an article. The authors have granted ASM a non-exclusive, world-wide license to publish the Supplemental Material files. Please contact the corresponding author directly for reuse.
