# Supplementary material for: Engineering the hyperthermophilic archaeon Pyrococcus furiosus for 1-propanol production
Source: Appl Environ Microbiol. 2025 Apr 7;91(5):e00471-25. doi: 10.1128/aem.00471-25 (PMC12094016; doi:10.1128/aem.00471-25)
Supplement: Supplemental material — Table S1 and Figures S1 to S6. [file aem.00471-25-s0002.pdf]

Appl. Environ. Microbiology

Engineering the Hyperthermophilic Archaeon *Pyrococcus furiosus* for 1-Propanol Production

Hailey C. O'Quinn[a], Jason L. Vailionis[b], Tania N. N. Tanwee[a], Katherine S. Holandez-Lopez[a,*], Ryan G. Bing[c], Farris L. Poole[a], Ying Zhang[b], Robert M. Kelly[c] and Michael W. W. Adams[a,#]

Department of Biochemistry and Molecular Biology, University of Georgia, Athens, GA 30602, USA[a]
Department of Cell and Molecular Biology, College of the Environment and Life Sciences, University of Rhode Island, Kingston, Rhode Island, USA[b]
Department of Chemical and Biomolecular Engineering, North Carolina State University, Raleigh, NC 27695-7905, USA[c]

Supplementary Information

Supplementary Table S1

Supplementary Figures S1-S6

Data File S1

Table S1. Primers used in this study

Primer ID	Primer Target	Direction	5'to 3' sequence
HC001	5' AdhA (TX514_0564)	Forward	GGA GGT TTG AAG GTG TGG GAA ACA AAA ATA AAT CCA AAT AAG
HC002	3' AdhA (TX514_0564)	Reverse	CCT AAA AAA GAT TTT AGA AAG ATT CTT CAT AAA TCT TGG C
HC003	5' *Pfu* genome region 1 D.S. flank	Forward	GCC AAG ATT TAT GAA GAA TCT TTC TAA AAT CTT TTT TAG GGG CAA CAA TCG
HC004	5' E6 (Msed_1426)	Reverse	GTT TCC CAC ACC TTC AAA CCT CCT TAC GTG GTA AGG AGT ACT TTC CC
HC005	Plasmid backbone (pGL006)	Reverse	ATG GCG GAG CAG ACG CTC GT
HC006	Plasmid backbone (pGL006)	Forward	AGA AGA GCG ACT TCG CGG AG
HC007	5' AdhF deletion cassette	Forward	GGA GCC TGG CAA CCT TAT G
HC008	3' AdhF deletion cassette	Reverse	CAC TTG CGA CAT TGG GCA G
SP2.017	3' *Pfu* genome region 1 D.S. flank	Reverse	TCG AGG AGT ACA ACA TCT TTG C
SP2.018	5' *Pfu* genome region 1 U.S. flank	Forward	GTT TGG GTT GTG AGA GAA AGC
SP2.054	5' P_{slp} promoter region	Forward	TAG ATA TTA TCG CAA ACA CCG
SP2.055	3' P_{slp} promoter region	Reverse	TTT TCT CCA CCT CCC AAT AAT C
SP.260q	E1α (Msed_0407) qPCR primer	Forward	CAC AAG CCT TCG GAC GAT C
SP.261q	E1α (Msed_0407) qPCR primer	Reverse	AGA TGG TGC CTC CTC TAT CAA C
SP.262q	E1β (Msed_0408) qPCR primer	Forward	CAG AGA GAG TTA CAG AGC TTC C
SP.263q	E1β (Msed_0408) qPCR primer	Reverse	TGT CCC TTA TTC ACC GCA TC
SP.264q	E1γ (Msed_1375) qPCR primer	Forward	CCT ACC CTC TAA CAA CAT GGA G
SP.265q	E1γ (Msed_1375) qPCR primer	Reverse	CCG TTG TCC ACA GTC CTG
SP.266q	E2 (Msed_0709) qPCR primer	Forward	AGA ACT TCA AGA TGA GCG GTG
SP.267q	E2 (Msed_0709) qPCR primer	Reverse	GTT ATC TCC TTC ACG GTC TTG G

SP.268q	E3 (Msed_1993) qPCR primer	Forward	GTT CAG AAG GCA ATG TCC AAG
SP.269q	E3 (Msed_1993) qPCR primer	Reverse	CTG TCC AGC TTC TCC ATG AG
SP.157q	E4 (Msed_1456) qPCR primer	Forward	CGG TTG TCA TTT ACG AGA GC
SP.158q	E4 (Msed_1456) qPCR primer	Reverse	CTG ATG GAC GAG AGA TCG TG
SP.066q	E5 (Msed_2001) qPCR primer	Forward	GCA GAA GCC TGG AAA TTC TC
SP.067q	E5 (Msed_2001) qPCR primer	Reverse	CGC TGC GAT CCT TAT ATC AC
SP.068q	E6 (Msed_1426) qPCR primer	Forward	GTT TCT CTC CAG GAG ACA GAG
SP.069q	E6 (Msed_1426) qPCR primer	Reverse	AGA CTG GTT ACC TTC ACC TTG
SP.375q	AdhA (Teth514_0564) qPCR primer	Forward	TCA TAC CTG AAA AAG CTG CTC C
SP.376q	AdhA (Teth514_0564) qPCR primer	Reverse	GGG TCA TCT ATG GCA TAC ATA GG
GL313q	AOR (PF0346) qPCR primer	Forward	CAT CAA AGA CGA GCA CAT TGA G
GL314q	AOR (PF0346) qPCR primer	Reverse	GCG AAC TTG ACC AAA TTC TCA C
SP.211q	ACSIα (PF1540) qPCR primer	Forward	ACA TGG AAG GTG TGA AAG ATG G
SP.212q	ACSIα (PF1540) qPCR primer	Reverse	ACT TTG TCA CTA CCT GCA AGA G
SP.213q	ACSIβ (PF1787) qPCR primer	Forward	CAT CAA GAA TGA CGA GGA AGC
SP.214q	ACSIβ (PF1787) qPCR primer	Reverse	AGG TCC AAA CTG TGG ATC TC
SP.215q	ACSIIα (PF0532) qPCR primer	Forward	AGA CAC CCA GGA AGA CAA G
SP.216q	ACSIIα (PF0532) qPCR primer	Reverse	GAA CCA GTA TGA CTT GCT GC
SP.217q	ACSIIβ (PF1837) qPCR primer	Forward	GAT GCA GAG ATT TTT GGT GTC C
SP.218q	ACSIIβ (PF1837) qPCR primer	Reverse	CTT GCA TCT TTT TCT GTG ATT GG
SP2.056q	SLP (PF1399) qPCR primer	Forward	GGA TGT TGA AGT TAC CGA CG
SP2.057q	SLP (PF1399) qPCR primer	Reverse	CTA CAA AGT CTG ATC CGT TCC
P669q	PORγ (PF0971) qPCR primer	Forward	CTG CTG GCA TGA GAT TGC
P670q	PORγ (PF0971) qPCR primer	Reverse	GAT CAA GTC TAG AGC CTC TTG G

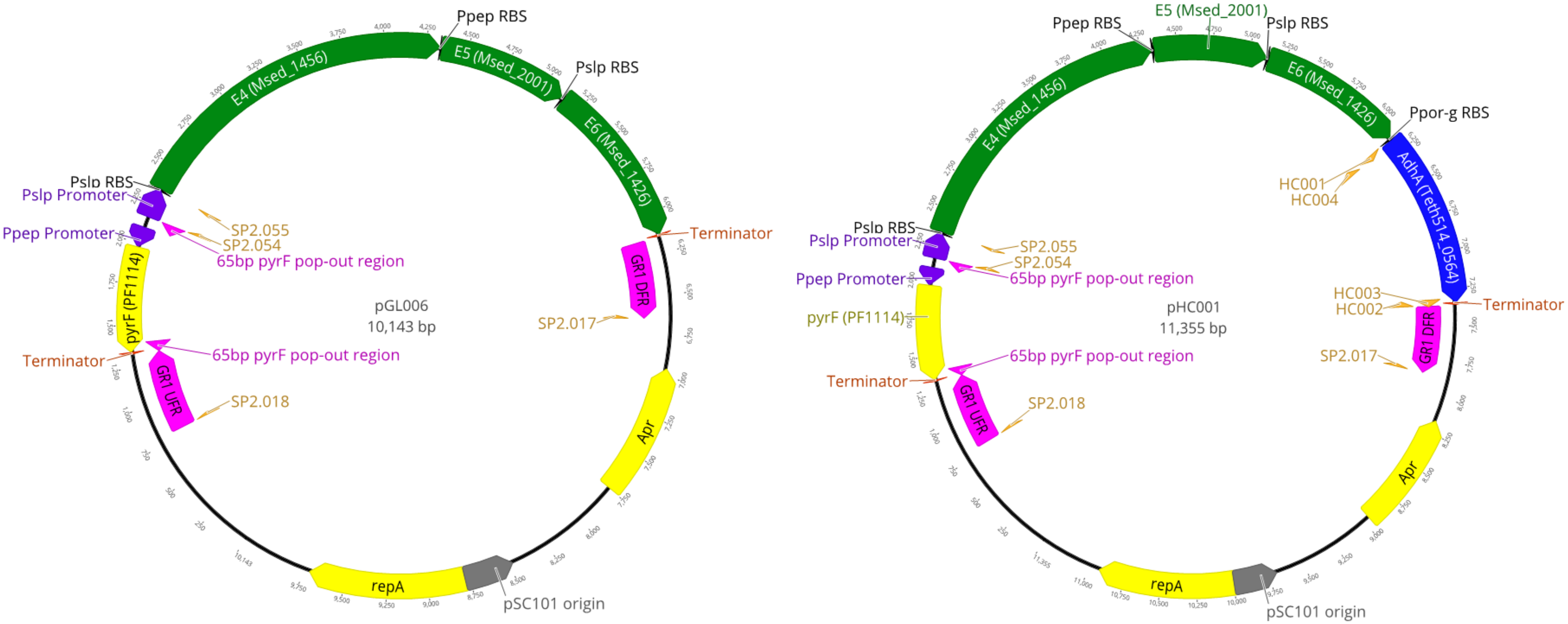

Figure S1. Plasmid maps of pGL006 and pHC001. pGL006 was used to generate pHC001 by adding Teth514_0564 (AdhA) to the existing operon. pHC001 encodes the expression of Msed_1456, Msed_2001 (E5), Msed_1426 (E6), and Teth514_0564 (AdhA). For integration into the *P. furiosus* genome at the PF0265-PF0266 intergenic region (genome region 1), 0.5 kb upstream and downstream flanking regions (UFR and DFR) border the operon.

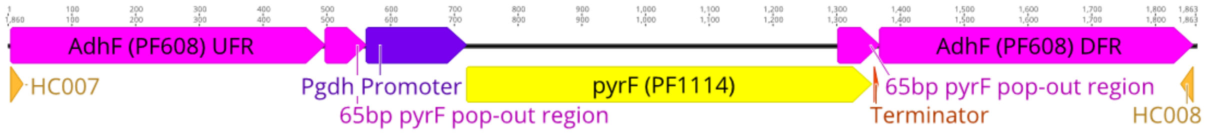

Figure S2. Construct for the deletion of the native *P. furiosus* AdhF (PF608). Upstream flanking region (UFR) and downstream flanking region (DFR) allow for integration of construct into genome.

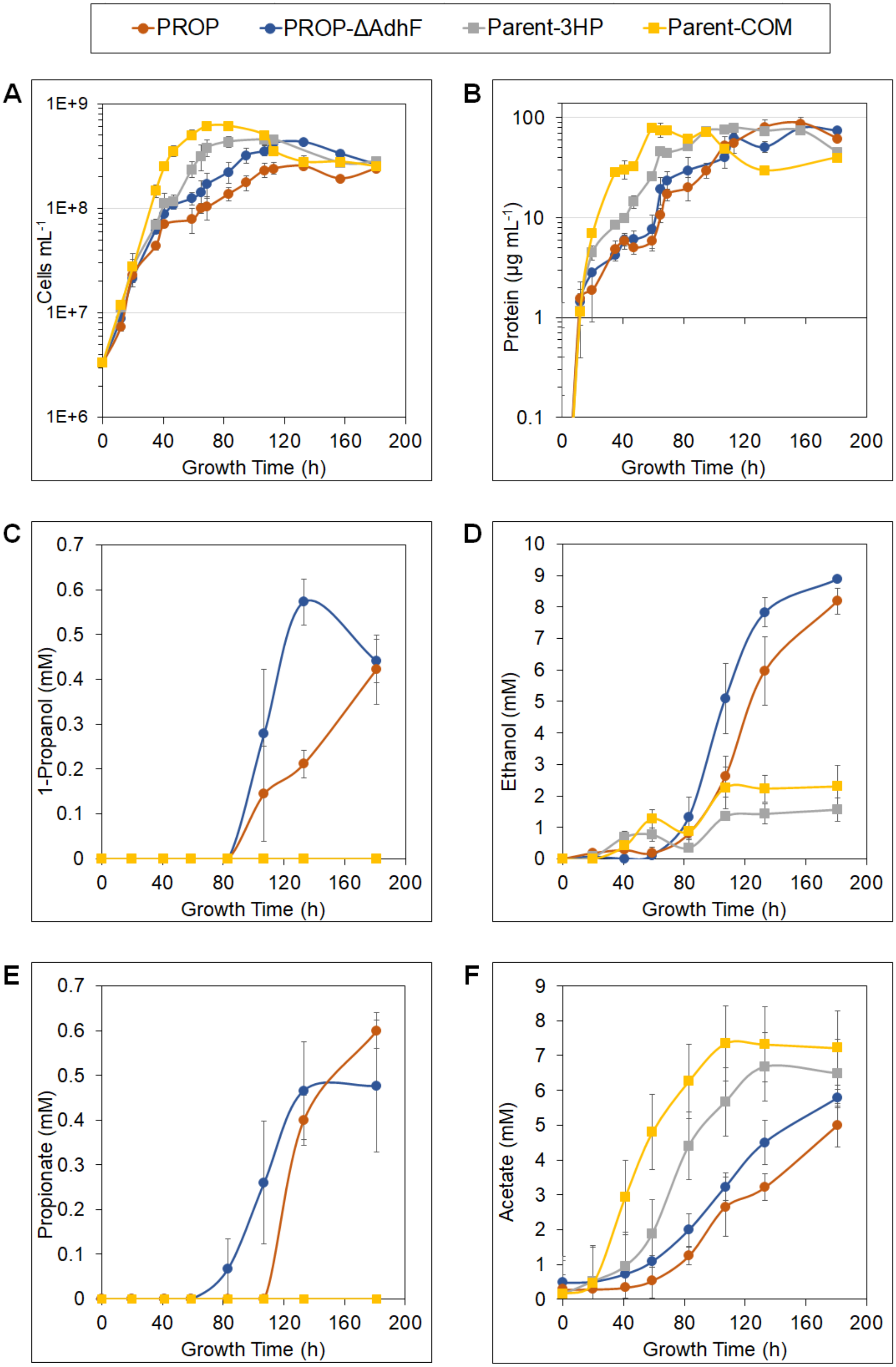

PROP
PROP-ΔAdhF
Parent-3HP
Parent-COM
A
Cells mL-1
1E+9
1E+8
1E+7
1E+6
0 40 80 120 160 200
Growth Time (h)
B
Protein (µg mL-1)
100
10
1
0.1
0 40 80 120 160 200
Growth Time (h)
C
1-Propanol (mM)
0.7 0.6 0.5 0.4 0.3 0.2 0.1 0
0 40 80 120 160 200
Growth Time (h)
D
Ethanol (mM)
10 9 8 7 6 5 4 3 2 1 0
0 40 80 120 160 200
Growth Time (h)
E
Propionate (mM)
0.7 0.6 0.5 0.4 0.3 0.2 0.1 0
0 40 80 120 160 200
Growth Time (h)
F
Acetate (mM)
9 8 7 6 5 4 3 2 1 0
0 40 80 120 160 200
Growth Time (h)

Figure S3. Growth of 1-propanol and control strains at 75°C. Growth of PROP (orange circles), PROP-ΔAdhF (blue circles), Parent-3HP (gray squares) and Parent-COM (yellow squares) are shown. A growth temperature of 75°C was maintained throughout. Error bars represent standard deviation; n=3 for each strain. A: Cell count in mg/mL of culture; B: protein concentration in μg/mL; C: 1-propanol, D: ethanol, E: propionate, and F: acetate concentration in mM present in spent media.

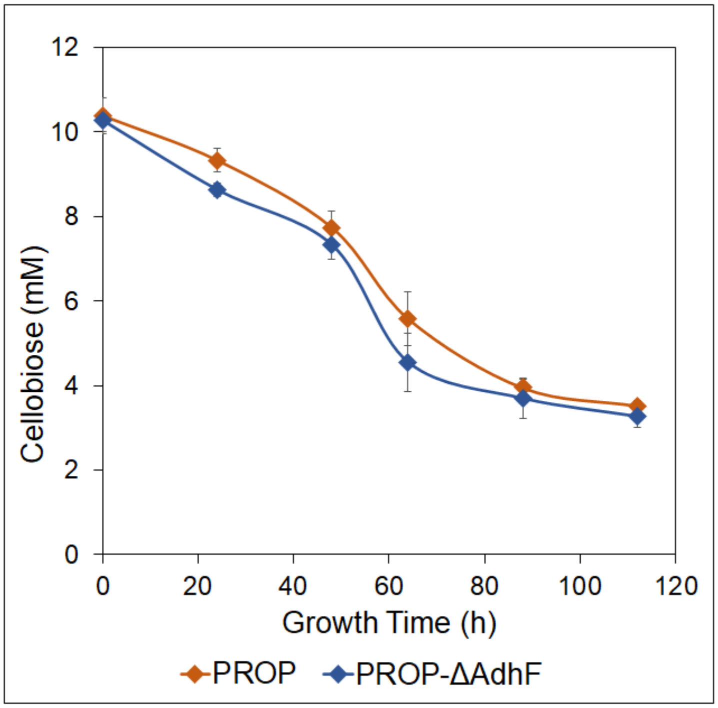

Figure S4. Cellobiose utilization of PROP (orange diamonds), PROP-ΔAdhF (blue diamonds) during temperature shift growth (98°C-75°C). A growth temperature of 98°C was maintained until cell count reached 1e+08 (hour 7) then maintained at 75°C. Error bars represent standard error; n=4 for each strain.

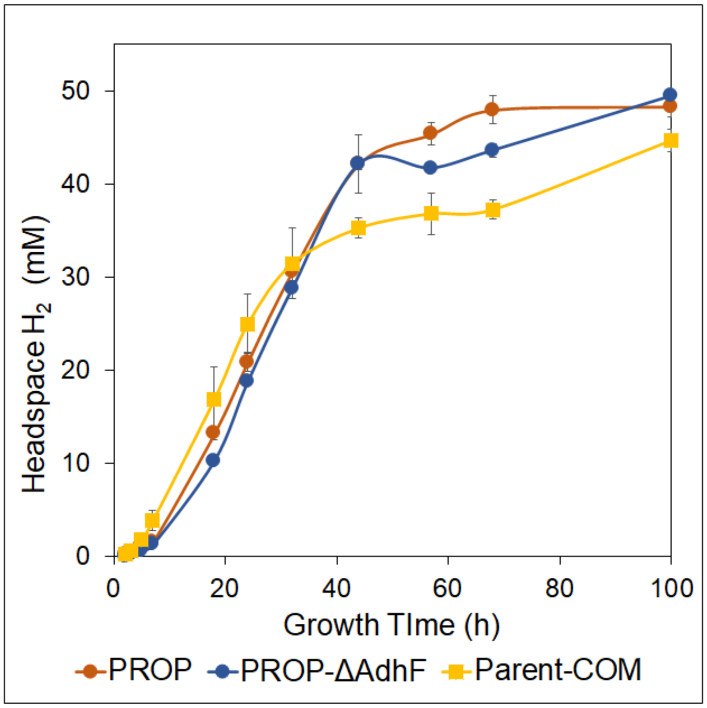

Figure S5. Hydrogen produced during closed bottle growth. Headspace H_2 for strains PROP-ΔAdhF (blue circles), PROP (orange circles), and Parent-COM (yellow squares) during a 100 hour temperature shift growth (98°C-75°C). Hydrogen concentration in sealed bottle headspace is shown in mM. Error bars represent standard error, n=3.

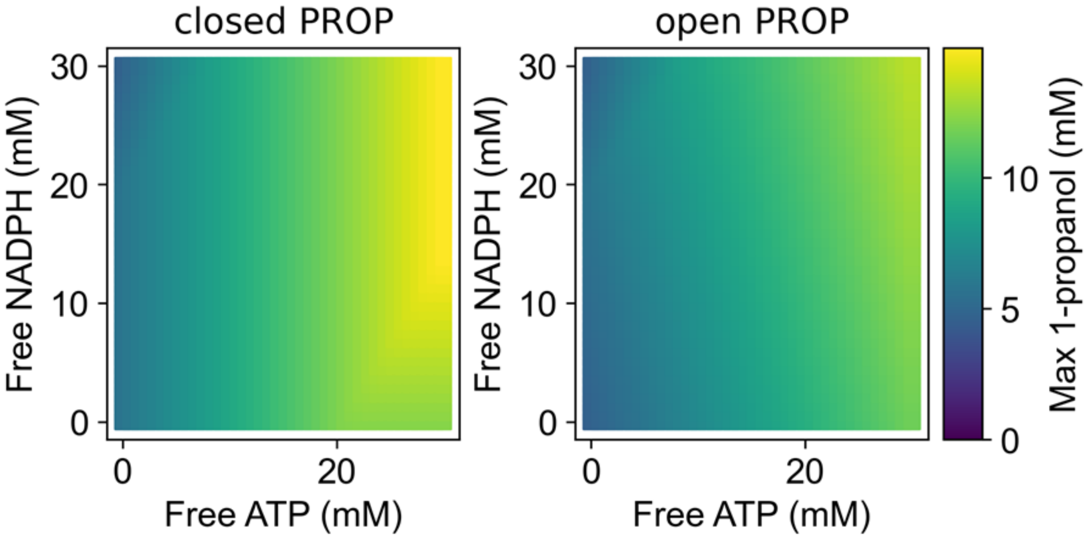

Figure S6. Theoretical maximum 1-propanol yield under varying levels of free ATP or NADPH in the closed Prop model.

Data File S1. Maximum propanol yields predicted by the PROP model when individual reactions were constrained based on the median of randomly sampled flux distributions.

This is supplied as a separate Excel file.
